# Acidic bile salts induces mucosal barrier dysfunction through *let-7a* reduction during gastric carcinogenesis after *Helicobacter pylori* eradication

**DOI:** 10.18632/oncotarget.24725

**Published:** 2018-04-06

**Authors:** Yasushi Takahashi, Kaname Uno, Katsunori Iijima, Yasuhiko Abe, Tomoyuki Koike, Naoki Asano, Kiyotaka Asanuma, Tooru Shimosegawa

**Affiliations:** ^1^ Division of Gastroenterology, Tohoku University, Miyagi, Japan; ^2^ Department of Gastroenterology, Akita University, Miyagi, Japan; ^3^ Department of The Second Internal Medicine, Yamagata University, Miyagi, Japan

**Keywords:** gastric cancer, helicobacter pylori eradication, mucosal barrier dysfunction, epithelial-mesenchymal transition

## Abstract

Gastric cancer (GC) after eradication for *Helicobacter pylori* (*H.pylori*) increases, but its carcinogenesis is not elucidated. It is mainly found in acid non-secretion areas (ANA), as mucosal regeneration in acid secretory areas (AA) after eradication changes the acidity and bile toxicity of gastric juice. We aimed to clarify the role of barrier dysfunction of ANA by the stimulation of pH3 bile acid cocktail (ABC) during carcinogenesis. We collected 18 patients after curative endoscopic resection for GC, identified later than 24 months after eradication, and took biopsies by Congo-red chromoendoscopy to distinguish AA and ANA (UMIN00018967). The mucosal barrier function was investigated using a mini-Ussing chamber system and molecular biological methods. The reduction in mucosal impedance in ANA after stimulation was significantly larger than that in AA, 79.6% vs. 87.9%, respectively. The decrease of zonula occludens-1 (ZO-1) and *let*-7a and the increase of snail in ANA were significant compared to those in AA. In an *in vitro* study, the restoration of ZO-1 and *let*-7a as well as the induction of snail were observed after stimulation. High mobility group A2 (HMGA2)-snail activation, MTT proliferation, and cellular infiltration capacity were significantly increased in AGS transfected with *let*-7a inhibitor, and *vice versa*. Accordingly, using a mini-Ussing chamber system for human biopsy specimens followed by an *in vitro* study, we demonstrated for the first time that the exposure of acidic bile salts to ANA might cause serious barrier dysfunction through the *let*-7*a* reduction, promoting epithelial-mesenchymal transition during inflammation-associated carcinogenesis even after eradication.

## INTRODUCTION

Prevalence and mortality rates of gastric cancer (GC) remain high throughout the world. Chronic inflammation due to *Helicobacter pylori* (*H. pylori*) is closely involved in gastric carcinogenesis, which consists of a multi-step process from chronic gastritis to intestinal metaplasia and gastric dysplasia/neoplasia [1, 2]. Although eradication treatment for *H. pylori* is recently widespread, its preventive effect on GC occurrence is uncertain [3–5]. In fact, the number of patients suffering from GC even after successful eradication (*i.e.*, post-eradication GC) is on the rise. Thus remains the urgent task to elucidate gastric carcinogenesis after elimination of the *H. pylori* component.

Previous studies investigated the histological changes using the updated Sydney system more than 10 years after eradication, and demonstrated that, although active inflammation was rarely seen within 1 year, chronic inflammation, atrophy, and intestinal metaplasia had persisted for a long time [6]. Another study revealed that the numbers of methylated oncogenic genes increased significantly in the gastric tissue with chronic inflammation [7]. These suggested that chronic inflammation persisting even after successful eradication might play a major role in the development of gastric carcinogenesis [3].

In our studies using Congo-red chromoendoscopy (CRE) to thoroughly ascertain the acid secretion ability in non-neoplastic gastric mucosa, we clearly identified the distribution of acid secretory areas (AA) and acid non-secretory areas (ANA) and demonstrated the following results. After eradication, AA became gradually spread from the greater curvature to the lesser curvature of the stomach [8], but there still remained the ANA, where atrophy, intestinal metaplasia, and chronic inflammation were histologically remnant, and where the occurrence rate of post-eradication GC was extremely high [9]. Ji *et al*. performed *in vivo* inspection for gastric antral mucosa using confocal laser endoscopy before and at 6 months after eradication in 42 *H. pylori*-positive patients, and they reported that the paracellular gap in metaplastic mucosa remained impaired, while those in non-metaplastic mucosa had cured [10]. These suggested that severe atrophic mucosa remaining after eradication might have a high carcinogenic risk, but it has not been elucidated how the inflammation-associated carcinogenic process occurs in atrophic mucosa after eradication.

Mucosal tissues in the digestive tract work as a physiological barrier against the invasion of external pathogens from the lumen. Regarding the mechanism of barrier dysfunction in *H. pylori*-positive patients, damage to cell-to-cell attachment, including tight junction (TJ) and adherence junction (AJ), was reported to be more remarkable than direct damage to epithelial cells by bacterial components [11]. The expressions of TJ/AJ, made up of complexes such as claudin, occludin, zonula occludens-1 (ZO-1), and E-cadherin, are decreased in epithelial-mesenchymal transition (EMT) during the carcinogenic or wound healing process [12, 13]. Accordingly, chronic inflammation in the gastric mucosa is likely prolonged by exposure to external stimulants that can pass through the paracellular gaps, resulting in worsening of the mucosal barrier dysfunction.

As for the components of gastric juice, the pH level in *H. pylori*-positive inflamed mucosa is about 6.8, while it is improved to 3–4 after eradication [14]. Actually, the ionized/non-ionized form of bile acid (BA) varies in a pH dependent manner [15]. In low pH conditions, most of the BA exists in a non-ionized form, which tends to permeate cell membranes more easily and to cause intracellular/paracellular damage more efficiently than the ionized form [4]. Therefore, we aimed to clarify the role of barrier dysfunction of the human gastric mucosa of ANA by stimulation with a pH3 bile acid cocktail (ABC) during the development of gastric carcinogenesis after the elimination of *H.pylori* bacterial components.

## RESULTS

### Patients’ background

The demographics of the subjects are shown in Supplementary Table 1. Mean age of them was 74.0 (7.6 S.D.) years with a male/female ratio of 15/3. The mean of follow-up duration after eradication therapy was 74.8 (51.5 S.D.) months, and the extension of corpus atrophy in the CRE findings were closed type 1–2/closed type 3-open type1/open type 2–3 for 3/7/8 patients, respectively. Most of the macroscopic findings of post-eradication GC were the depressed type with a tumor diameter of 12.5 (13.9 S.D.) mm, and the majority of their pathological findings were well-moderately differentiated adenocarcinoma limited to the mucosal layer, classified as T1N0M0 (mucosal cancer: submucosal cancer = 15:3) [16].

### Mucosal impedance and permeability in a mini-ussing chamber system

**Figure 1:**
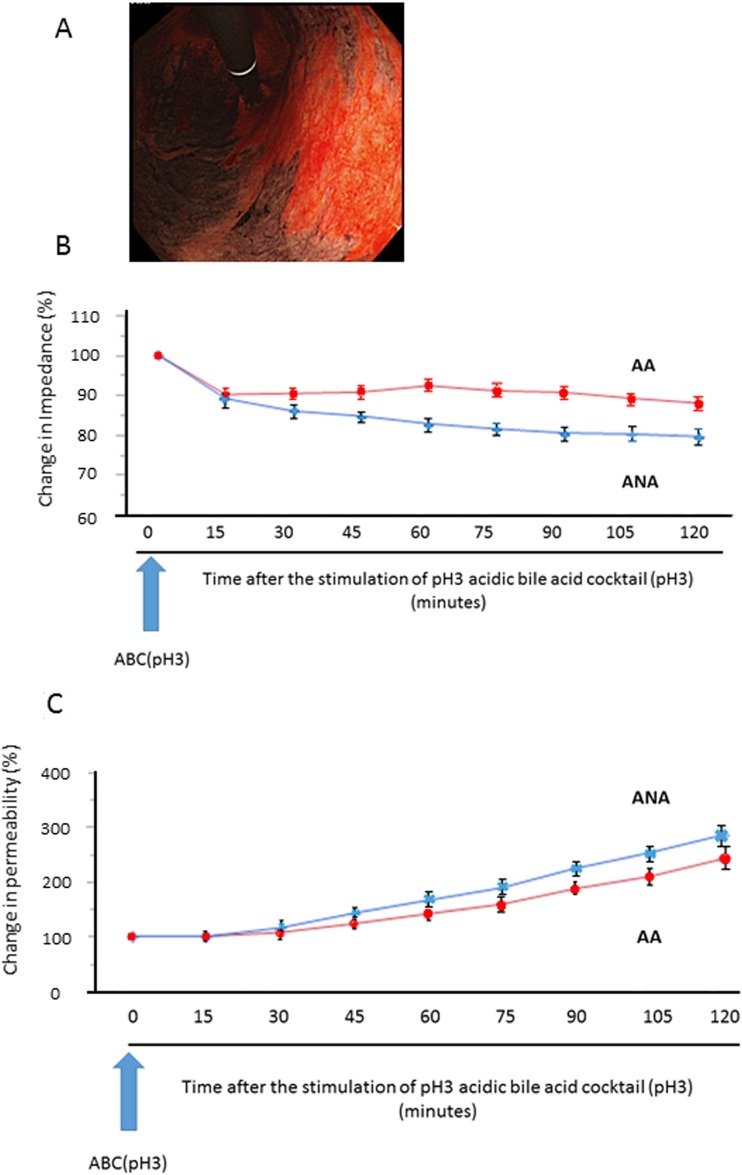
Sequential change of mucosal barrier function after stimulation with acidic bile cocktail (ABC) in a mini-Ussing chamber system (**A**) As a representative image, we clearly identified the distribution of AA (black) and ANA (red) by Congo-red chromoendoscopy, whose color is changed due to the reaction with gastric acid, and biopsy samples were taken from each area. (**B**) Mucosal impedance decreased gradually for 120 minutes immediately after the ABC stimulation, and its decrease in the specimens from ANA (blue) was significantly greater than in those from AA (red) (*P* = 0.043, *N* = 18, mixed effects model). (**C**) Mucosal permeability increased with time, and its increase in the specimens from ANA (blue) tended to be larger than that from AA (red), despite the lack of significant difference (*P* = 0.309, mixed effects model).

Mucosal impedance decreased gradually for 120 minutes immediately after the stimulation, and its decrease in the specimens from ANA was significantly larger than in those from AA (Figure 1B. 79.6% vs. 87.9%: *N* = 18, *P* < 0.05, mixed effects model). Mucosal permeability after the stimulation increased with time, and the increase in the specimens from ANA tended to be larger than in those from AA, despite the lack of significant difference (Figure 1C. 303.3% vs. 257.6%: *N* = 18, *P* = 0.309, mixed effects model).

### Pathological evaluation of gastric mucosa in ANA and AA

Based on the updated Sydney system, the score of activity was zero in both areas, but the scores of inflammation, atrophy, and metaplasia in the specimens from ANA were significantly higher than in those from AA (Supplementary Table 2, *P* < 0.0001).

### TJ gene expressions in ANA and AA

**Figure 2:**
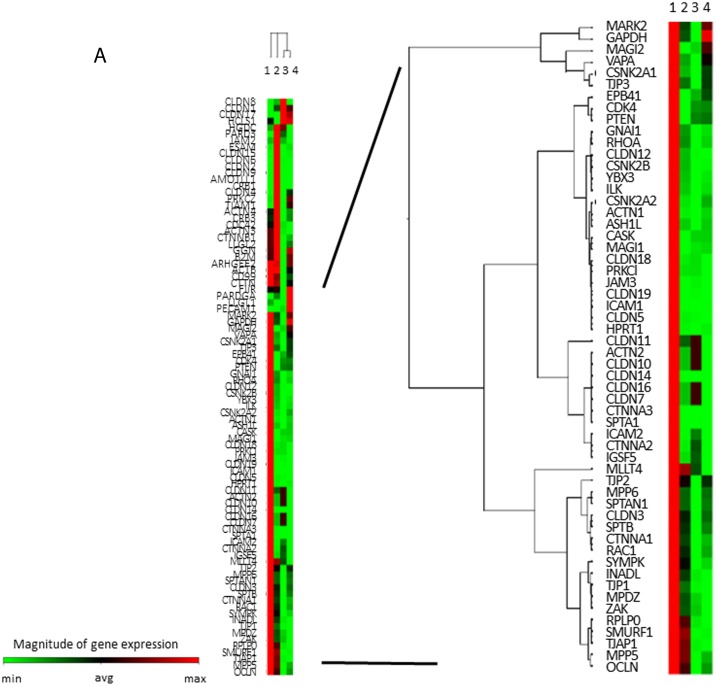

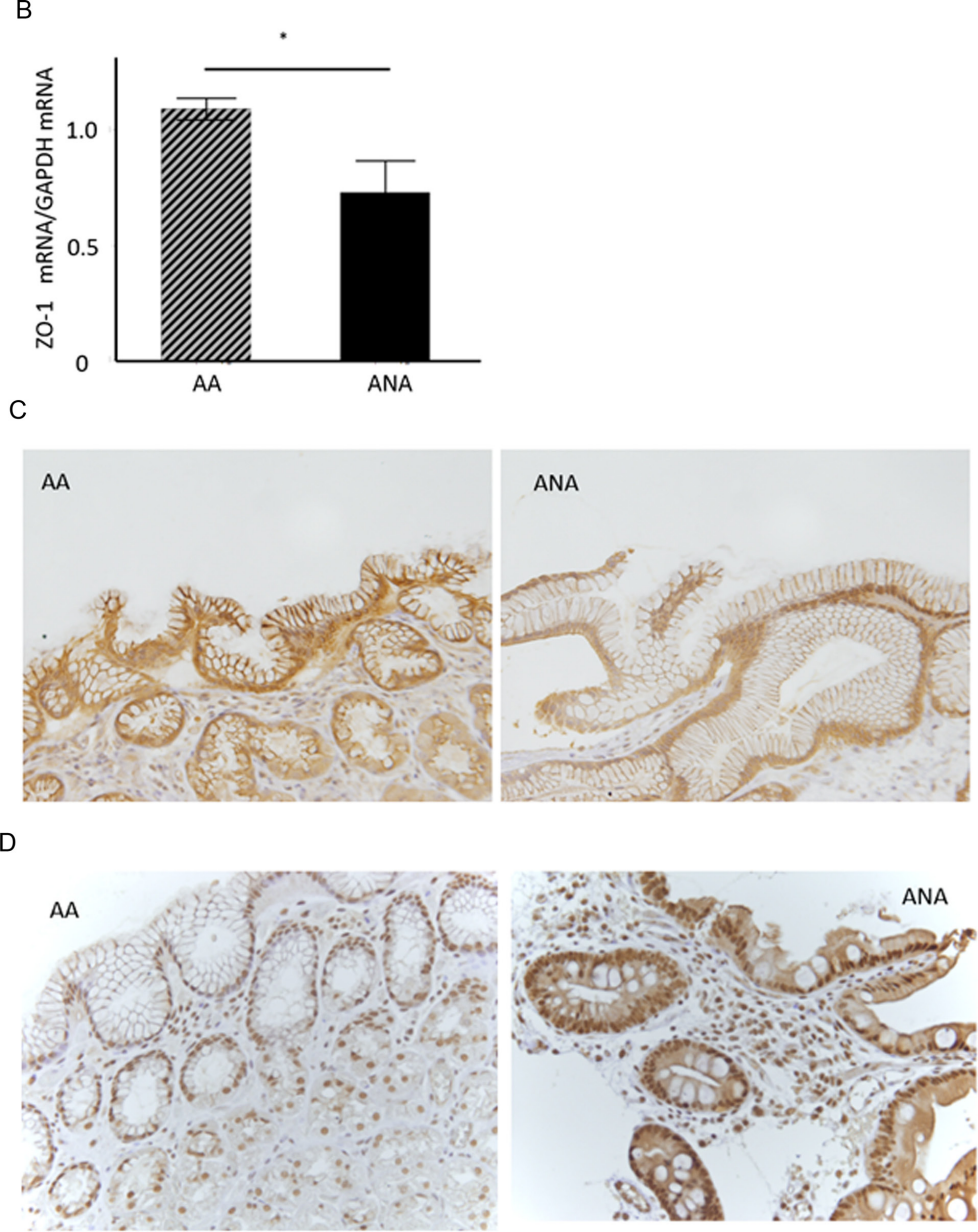
TJ-gene expressions in ANA and AA (**A**) In a comprehensive analysis of the expressions of TJ-genes as a discovery phase, frozen-stocked specimens from post-eradication mucosa (1. AA, 2. ANA) and *H. pylori*-infected mucosa (3. AA, 4. ANA) were studied using a human tight junction genes RT^2^ PCR array analysis (*N* = 3–5). (**B**) In the validation phase, the expression of ZO-1 mRNA in ANA was significantly diminished compared to that in AA (*N* = 18, ^*^P = 0.008, Student`s *t*-test). (**C**, **D**) A representative image of an immunohistochemical study for ZO-1 and snail (×400). The expression of ZO-1 in ANA (C: right) was decreased in comparison to that in AA (C: left), although that of snail in ANA (D: right) was increased in comparison to that in AA (D: left).

To comprehensively analyze the expressions of TJ genes as a discovery phase, frozen-stocked specimens from post-eradication mucosa and *H. pylori*-infected mucosa were studied using a human tight junction genes RT^2^ PCR array analysis (*N* = 3–5). According to the data of the mucosal barrier dysfunction, 56 candidate genes were selected among the TJ genes whose expressions were markedly decreased in ANA and, meanwhile, attention was focused on ZO-1 (TJP1) and claudin-12 (Figure 2A).

As a validation phase, the expression of ZO-1 mRNA in the samples from ANA was significantly diminished compared to those from AA (Figure 2B, ZO-1mRNA/GAPDH mRNA: 0.78 (0.49 S.D.) vs. 1.09 (0.17 S.D.): *N* = 18, *P* = 0.008, Student's *t*-test). An immunohistochemical study demonstrated that the scoring index of ZO-1 protein in ANA was significantly decreased compared to those in AA (Figure 2C, low expression/high expression: ANA vs. AA = 17/1 vs. 10/8, *P* = 0.007, Chi-square test). The expression of claudin-12 protein in ANA was also lower than that in AA (data not shown), while the expression of snail protein in ANA was higher than that in AA (Figure 2D). From these investigations using *ex vivo* human specimens, it became evident that mucosal barrier damage of ANA was markedly greater than that of AA.

### Effect of exposure of ABC to TJ and EMT gene expressions

**Figure 3:**
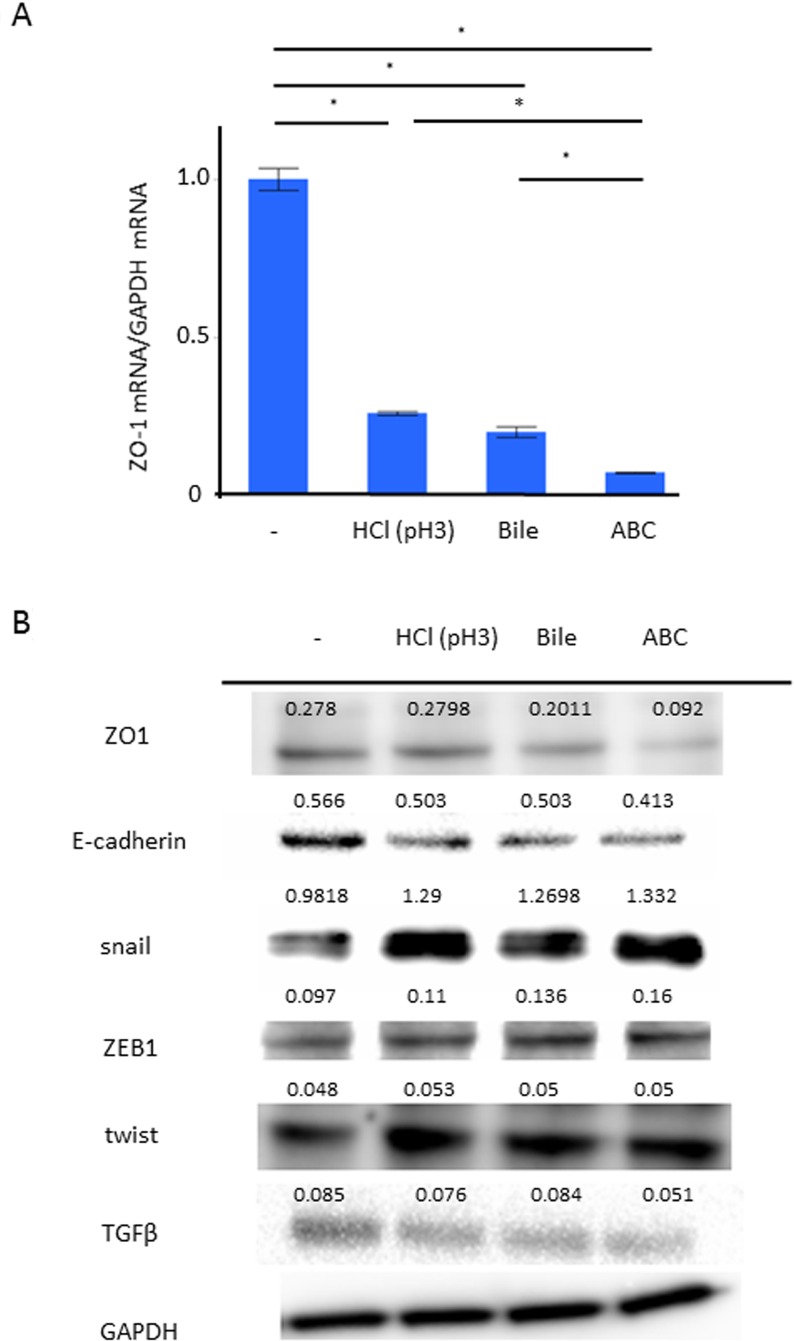
Effect of the ABC exposure to gene expressions of TJ/AJ and EMT The expression of ZO-1 in AGS with the 24-hour stimulation of weak acid, bile acid, and ABC was investigated. (**A**) RT-qPCR demonstrated that, compared to control, the expression of ZO-1 mRNA was significantly inhibited in the group stimulated by weak acid or bile acid, and even more significantly inhibited in the ABC-stimulated group (*P* < 0.05, *N* = 5, Turkey-Kramer HSD test). (**B**) The expressions of TJ/AJ proteins were investigated in Western blot analyses.

We applied AGS among the human cell-lines whose phenotype of TJ gene expression was similar to that of human gastric mucosal tissue after eradication (Supplementary Figure 1). The mRNA expression of ZO-1 in AGS with/without the 24-hour stimulation of weak acid, BA, and ABC was investigated by Reverse Transcription-quantitative Polymerase Chain Reaction (RT-qPCR). Compared to control, the expression of ZO-1 mRNA was significantly inhibited in the group stimulated by weak acid or BA, and even more significantly inhibited in the ABC-stimulated group (Figure 3A, *P* < 0.05, *N* = 5, Turkey-Kramer HSD test). The expressions of TJ/AJ proteins were investigated in Western blot analyses (Figure 3B). Compared to control, the protein expressions of ZO-1 and E-cadherin were significantly inhibited in the groups stimulated by weak acid or BA, and more significantly inhibited in the ABC-stimulated group. In view of the fact that the TJ/AJ expressions can be decreased during the EMT process, the expressions of ZEB1, snail, and twist were also studied in the barrier damage progression. Compared to control, the protein expressions of snail and ZEB1 were enhanced in the group stimulated by weak acid or BA, and more significantly inhibited in the ABC-stimulated group. In particular, the snail induction was markedly enhanced at the mRNA/protein levels, but the protein expression of TGF-β showed no increase with the stimulation.

### Identification of candidate microRNAs causing barrier dysfunction

**Figure 4:**
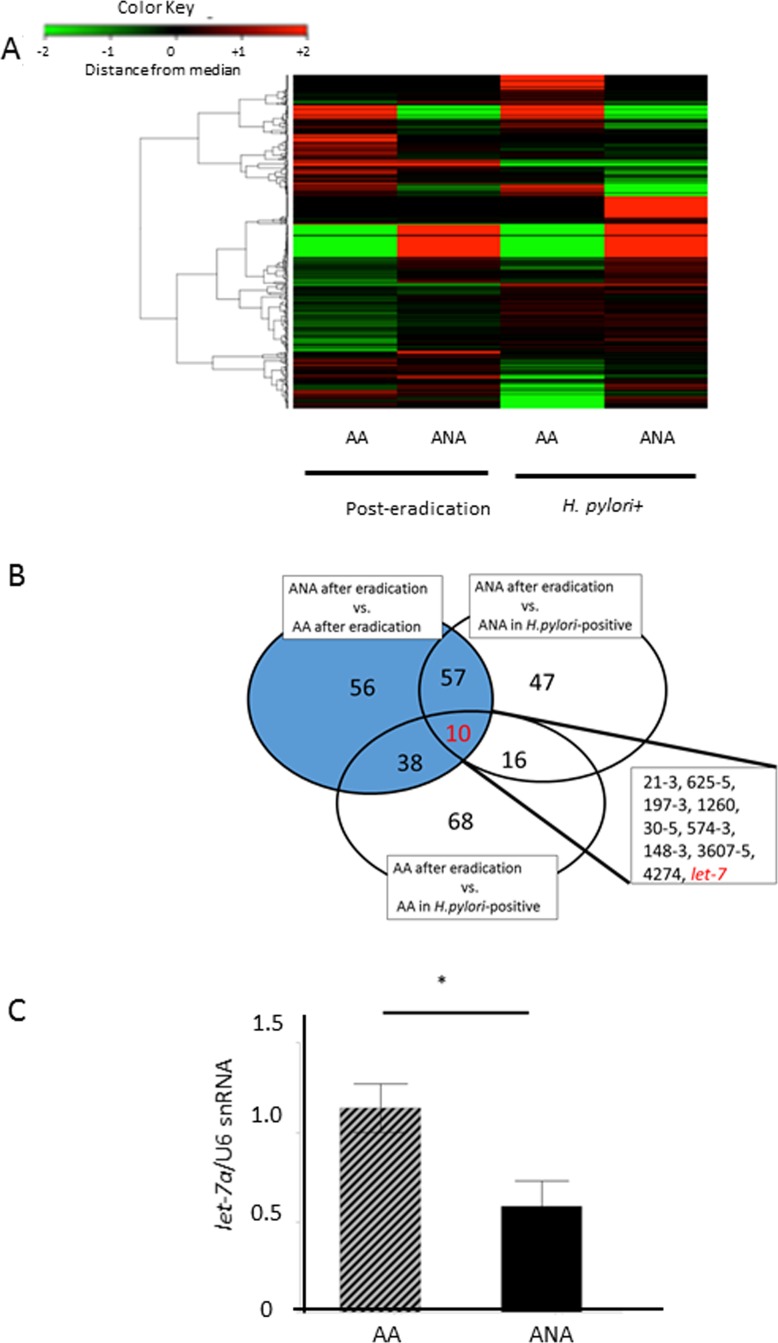
Identification of candidate microRNAs that cause barrier dysfunction (**A**) A heat-map of a Taqman microRNA microarray analysis in a discovery phase. There were significant differences in the microRNA profiles between samples from post-eradication mucosa and *H-pylori*-positive mucosa (*N* = 5). (**B**) 10 microRNAs were extracted as candidate miRNAs, including miR 21-3, miR 625-5, miR 197-3, miR 1260, miR 30-5, miR 574-3, miR 148-3, miR 3607-5, miR 4274, and *let*-7. Target microRNAs that yielded differences at least 2-fold or 0.5-fold expression levels were selected as candidate microRNAs., (**C**) In the validation phase, the expression of *let*-7a in ANA was significantly inhibited in comparison to AA (*N* = 18, *P* = 0.0032, Student`s *t*-test).

In the discovery phase, a Taqman microRNA microarray analysis demonstrated differences in the microRNA profiles between post-eradication mucosa and *H-pylori*-positive mucosa (*N* = 5, Figure 4A). Ten microRNAs were extracted as candidate microRNAs (Figure 4B, miR 21-3, miR 625-5, miR 197-3, miR 1260, miR 30-5, miR 574-3, miR 148-3, miR 3607-5, miR 4274, *let*-7). Of these, we focused on onco-suppressive *let*-7a and onco-genic mir21-3p, whose expressions were reported to change after eradication [17, 18]. In the validation phase, the expression of *let*-7a in ANA was significantly inhibited compared to AA (Figure 4C, *let*-7a/U6 snRNA: 0.59 (0.51) vs. 1.14 (0.49), *N* = 18, *P* = 0.0032, Student`s *t*-test), although that of miR-21-3p showed no significant difference (miR-21-3p/U6 snRNA: 2.27 (3.1) vs. 1.01 (0.01), *P* = 0.0573, Student`s *t*-test).

### Role of *let*-7a in barrier dysfunction by acidic bile *in vitro*

**Figure 5:**
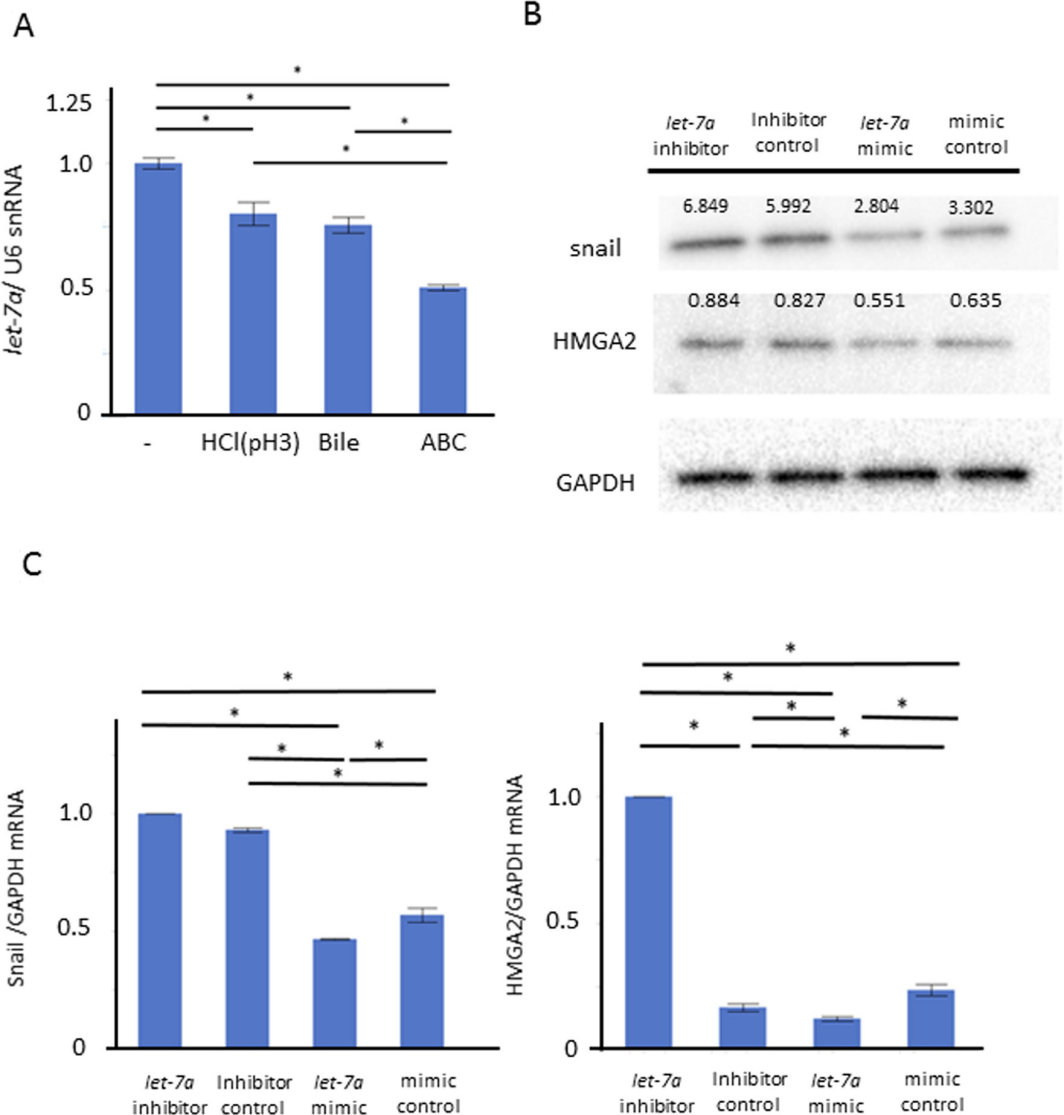
Role of *let-*7a in barrier dysfunction by ABC stimulation We performed an *in vitro* study using AGS to clarify the role of *let-7a* in maintaining the barrier function. (**A**) Compared to control, the *let*-7a expression was significantly inhibited in the group stimulated with weak acid or bile acid, and even further inhibited in the ABC-stimulated group (*N* = 6, *P* < 0.05, Tukey-Kramer test). (**B**) The plasmid of *let*-7a inhibitor, mimic, and the negative control were transfected in AGS to investigate the effect on the induction of HMGA2 as well as snail (*N* = 5). (**C**) Transfection with the *let*-7a inhibitor plasmid increased the expressions of HMGA2 and snail at the mRNA levels. Transfection with the *let-7a* mimic plasmid decreased their expressions (*N* = 5).

We performed a further *in vitro* study for the role of *let-7a* in maintaining the barrier function. Compared to control, the *let*-7a expression was significantly inhibited in the groups stimulated with weak acid or BA, and even further inhibited in the ABC-stimulated group (Figure 5A, *N* = 6, *P* < 0.05, Tukey-Kramer test). These demonstrated that the trend in the *let*-7a reduction with the stimulation could occur along with that in the expressions of ZO-1, E-cadherin and snail, suggesting that *let*-7a may be involved in the induction of these expressions. When we explored for target genes of *let*-7a using Target Scan 7.1, ZO-1, ZEB1, and High mobility group A2 (HMGA2), an upstream gene of snail, were found (http://www.targetscan.org). Considering that microRNA basically inhibits target genes at a post-transcription level in humans, we assumed that *let*-7a may inhibit the snail induction through the HMGA2 inactivation. Then, the plasmids of a *let*-7a inhibitor, mimic, and their negative control were transfected in AGS to investigate their effect on the induction of HMGA2 as well as snail. Transfection with a *let*-7a inhibitor plasmid increased the HMGA2 and snail expressions at mRNA and protein levels, while transfection with a *let-7a* mimic plasmid decreased them as well (Figure 5B–5C, *N* = 5).

**Figure 6:**
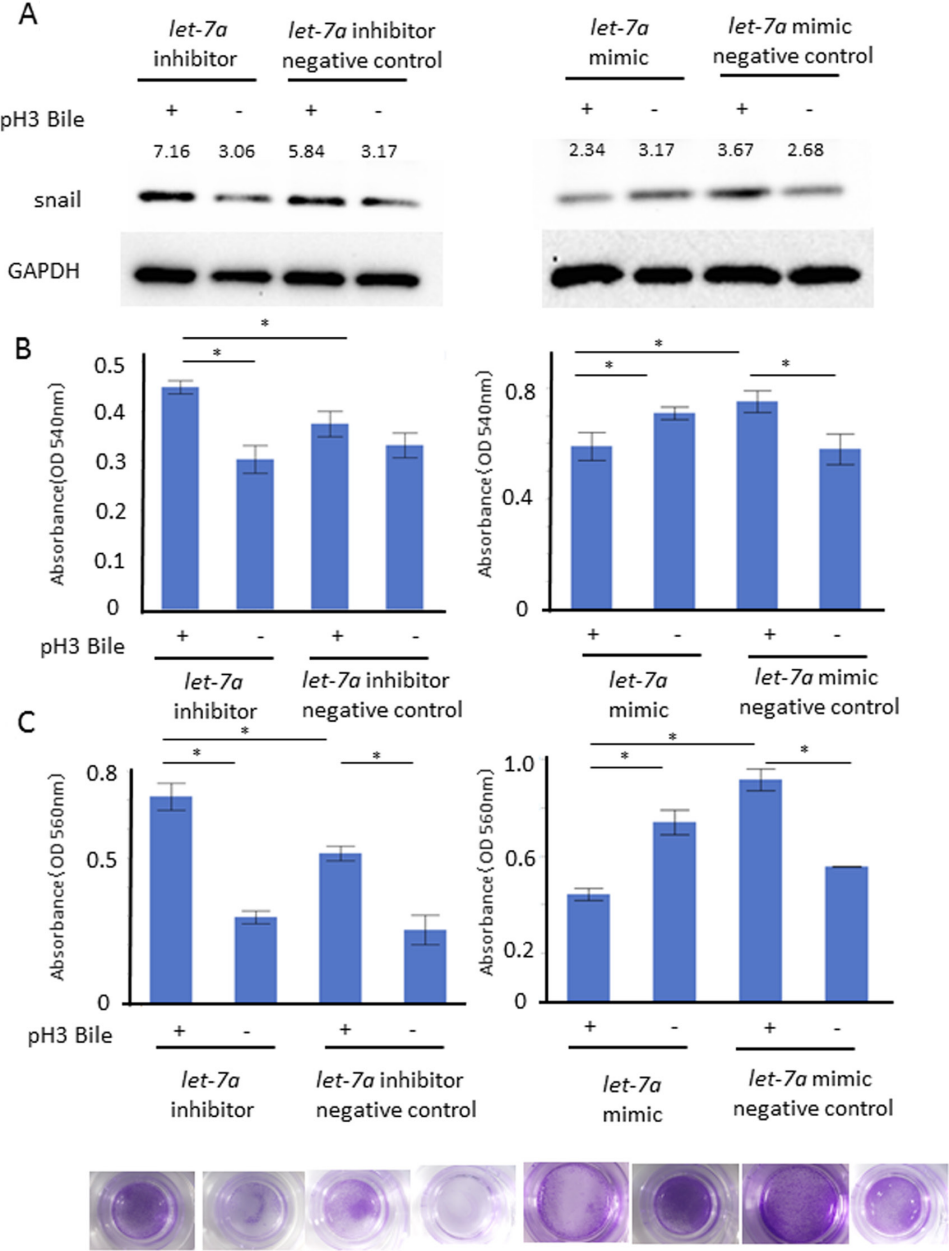
Functional role of *let-7a* in barrier dysfunction Cell proliferation and cell infiltration ability were investigated with/without the ABC stimulation on the AGS transfected with a *let-7a* inhibitor, mimic, negative control plasmids. The snail expression (**A**) as well as MTT cell proliferation (**B**) and cell infiltration ability (**C**) were enhanced under the *let*-7a inhibition, while they were inhibited by the *let*-7a induction (A) *N* = 5, (B) *P* < 0.05, *N* = 4, Tukey-Kramer test, (C) *P* < 0.05, *N* = 4, Tukey-Kramer test, representative photo of cellular infiltration assay).

To confirm the functional role of *let*-7*a* in EMT, we investigated the cell proliferation and cell infiltration ability with/without the ABC stimulation in these transfected cells. The snail expression (A) as well as the cell proliferation (B) and the cell infiltration ability (C) were enhanced in AGS transfected with a *let*-7a inhibitor, while they were inhibited in AGS transfected with a *let*-7*a* mimic (Figure 6A, *N* = 5, Figure 6B–6C, *P* < 0.05, *N* = 4, Tukey-Kramer test).

## DISCUSSION

Using a mini-Ussing chamber system with the ABC stimulation of human specimens, we demonstrated for the first time that the exposure of acidic bile salts might cause severe barrier dysfunction through the *let-7a* reduction in non-dysplastic gastric mucosa in ANA even after successful eradication. We also elucidated the functional role of *let-7a* in the barrier dysfunction by the ABC stimulation, which might promote EMT through the *let*-7*a* inhibition followed by the induction of HMGA2 and snail as a pivotal inducer of inflammatory carcinogenesis.

First, we discuss the characteristics of the subjects, whose samples were taken under the CRE finding later than 6 years after *H.pylori* eradication. In a long-term follow-up study by the CRE before and after eradication, we previously demonstrated that AA became gradually spread from the greater curvature to the lesser curvature of the stomach until 24 months after eradication and stopped thereafter [9]. Moreover, in contrast to the corpus atrophy observed by white-light endoscopy or autofluorescence endoscopy, corpus atrophy in the CRE findings was more strongly associated with the source of gastric carcinogenesis, especially after eradication [19]. Considering that the ectopic recurrence rate of GC after endoscopic resection was reported to be extremely high [20], it is suggested that even non-dysplastic mucosal tissue in ANA, the major target in this study, might maintain a malignant potential. Therefore, the subjects may be reasonable for investigating about the early stage of gastric carcinogenesis in ANA.

Next, we address about the validity of the ABC stimulation. The pH level of gastric juice recovers to approximately 3–4 after eradication, and the cellular toxicity of BA is dependent on the pH level [14, 15]. We demonstrated that the ABC stimulation induced greater barrier dysfunction and EMT than either acid or BA alone, consistent with previous studies [21]. Particularly, in such low pH conditions as the intra-gastric condition after eradication, BA can cause intracellular damage by dissolution of the cell membranes and TJs. Additionally, several studies demonstrated the carcinogenicity of BA itself, as follows: 1) in a rat model of duodeno-gastric reflux of BA through the pyloric ring, the incidence of GC was at a high rate of 41%; and 2) the concentration of BA in gastric juice retained in the stomach with atrophic mucosa is 100–1000 μM [22, 23]. The higher the concentration of BA in the gastric juice, the higher the occurrence of intestinal metaplasia and GC. Although the concentration of BA in the chamber system differed from that in the *in vitro* condition, both concentrations can be considered as physiological concentrations.

Third, we revealed the usefulness of a mini-Ussing chamber system for elucidating the role of acidic bile exposure in barrier dysfunction during gastric carcinogenesis after the elimination of *H. pylori*. Actually, the epithelial tissue of the digestive tract functions as a physiological barrier against external stimulants, and it is the breakdown of the TJ/AJ system that mainly causes barrier dysfunction, rather than cellular damage due to bacterial components [11]. A previous study reported that stimulation by *H.pylori* with/without interleukin (IL)-6 and IL-8 decreased the transepithelial electric resistance (TEER) and increased the fluorescein permeability in an EndOhm chamber model for human T84 cell-lines [24]. Instead, we applied the mini-Ussing chamber system, which can directly detect minute barrier damage of the *ex vivo* human mucosal tissues similar to that which occurs *in vivo* in the mucosal tissue with various cellular heterogeneity, although an *in vitro* study using cultured cell-lines cannot mimic the *in vivo* condition. Using a chamber system of esophageal mucosa with an explosion of nitric oxide species, Ito *et al*. reported a decrease of mucosal impedance and an enhancement of mucosal permeability, as well as damaged paracellular gaps at the electron microscopic level [25]. To date, the chamber system for human biopsy samples has been used to elucidate the etiology of several gastrointestinal diseases, such as Barrett's carcinogenesis, functional dyspepsia, and inflammatory bowel diseases [26–28]. In the present study in which human gastric mucosa was first applied for this chamber system, it became evident that the impairment of mucosal impedance in ANA by the acidic bile stimulation was significantly more severe than that in AA, despite the lack of a significant difference in fluorescein-detected permeability between them. In fact, previous studies demonstrated that the detection sensitivity for mucosal permeability could be affected by the molecular weight of the permeating substances [29]. Accordingly, change in the mucosal impedance enables a detailed investigation of the barrier dysfunction under conditions that mimic the *in vivo* intra-gastric environment after eradication.

Furthermore, we demonstrated that, together with impairment of the expressions of the TJ genes, the snail expression was markedly induced in ANA. EMT is involved not only in cancer invasion and metastasis but also in wound healing [30, 31]. Previous studies revealed that the snail expression in non-neoplastic mucosa around GC lesions was significantly increased compared to that in normal gastric mucosa [32]. Another investigation also showed that acetaldehyde induced snail expression, inhibited ZO-1 expression, and decreased TEER [12]. These findings suggested that EMT might play an important role in breaking the homeostasis of non-neoplastic tissue. We revealed that the ABC stimulation induced the expression of snail particularly among EMT markers, while it inhibited those of ZO-1 and E-cadherin, a major downstream target of the snail family [31]. Therefore, we targeted snail as one of central genes in the barrier function.

For the next step, we studied the microRNA expression in a TGFβ-independent pathway of the snail induction, because the TGF-β expression, a representative inducer of snail [32, 33], did not increase under the ABC stimulation. Using a microRNA microarray analysis followed by RT-qPCR, we demonstrated, using biopsy samples taken under the CRE findings, that the expression of onco-suppressive *let-*7a was more significantly inhibited in ANA than in AA. On the other hand, using biopsy specimens taken by white-light endoscopic findings, Matsushima *et al*. reported the increase of *let-7a* expression in gastric mucosal tissue after *H.pylori* eradication [17]. These discrepancies may result from the fact that CRE can more clearly classify the background gastric mucosa in view of the acid secretory function and histological features, compared to white-light endoscopy [19].

To elucidate the role of *let-*7*a* in the snail induction, further *in vitro* investigations using AGS, whose phenotype of TJ gene expressions was similar to that in human non-neoplastic gastric mucosa, were performed. To date, the onco-suppressive effect of *let-*7a against gastric carcinogenesis induced by *H.pylori* was reported to be exerted through the inhibition of the K-Ras family at the post-transcription level, and the inhibition of HMGA2 in cell proliferation, differentiation, self-renewal and EMT [17, 18, 31–36]. In this study, the mRNA and protein expressions of HMGA2 and snail were reversibly paralleled with the *let-7a* expression and, moreover, the cell proliferative/invasive ability was also increased by the ABC exposure, instead of *H.pylori*, in AGS transfected with a *let-7a* inhibitor plasmid and *vice versa*. Basically, the role of microRNA in humans is to inhibit gene expression at the post-transcription level, but recent studies demonstrated that *let*-7a inhibited the HMGA2 induction at the mRNA and protein levels in both human specimens and cell-lines [37]. Therefore, it is reasonable that the reduction of *let-7a* by the ABC stimulation might induce EMT through the activation of a HMGA2-snail cascade, along with the barrier dysfunction through the inhibition of ZO-1 and E-cadherin. These suggested that the reduction of *let-7a* in ANA might sustain the malignant potential even after *H.pylori* eradication.

The limitations of this study may be a selection bias due to the small numbers of subjects treated by curative endoscopic treatment for early GC, and the lack of sequential changes in molecular markers after eradication in each subject. However, such patients may still be suitable for the investigation of gastric carcinogenesis from the non-neoplastic mucosa of ANA. Another possible clinical issue in the use of CRE is its complicated procedure and possible toxicity shown in a murine study [38]. However, it is difficult to assess the possibility of adverse events for patients having only one session of CRE. Further, we routinely suctioned away residual dyes at the end of the procedure. On the other hand, the distribution of AA and ANA was patchy, especially after eradication, so the precise identification of biopsy sites proposed in the updated Sydney system may be difficult under a white-light endoscopic inspection [19]. With appropriate consideration for a balance between the risk and benefit of elucidating gastric carcinogenesis after eradication, we carefully identified ANA as the source of post-eradication GC, and used biopsy samples in the mini-Ussing chamber system with the ABC stimulation, which mimics the *in vivo* intra-gastric environment.

In conclusion, we demonstrated that serious damage of the mucosal barrier in ANA after eradication was triggered by the stimulation of acidic bile salts in a mini-Ussing chamber system, and that the reduction of onco-suppressive *let-7a* might promote EMT through the induction of a HMGA2-snail cascade in the process of such barrier dysfunction. Accordingly, even after successful eradication, acidic bile salts in the gastric juice might induce mucosal dysfunction with changes in the microRNA profile, which might drive the development of gastric carcinogenesis. Therefore, with the recent remarkable increase in the necessity of screening endoscopic examination after eradication, we should give more careful attention to areas of severe atrophic gastric mucosa for early detection of post-eradication GCs. Further studies can be expected to yield biomarkers for the identification of individuals with a high-risk of post-eradication GC in a risk-stratified screening strategy.

## MATERIALS AND METHODS

### Study subjects

The subjects were 18 patients who underwent endoscopic examination between August 2015 and July 2017 after curative endoscopic submucosal dissection for “post-eradication GC” lesions, which were newly discovered later than 24 months after successful eradication. In fact, the occurrence of ectopic GC is extremely high after endoscopic resection of primary GC [39]. The following exclusion criteria were applied: 1) patients younger than 20 years old or older than 85 years old; 2) patients with a history of chemotherapy, radiotherapy or upper abdominal surgery; 3) patients taking nonsteroidal anti-inflammatory drugs, antibiotics, or any acid secretion inhibitors; 4) patients with serious medical conditions; 5) patients suffering from mental disorders; 6) patients who might be pregnant; 7) patients with inappropriate specimens; 8) patients with contraindication for endoscopic biopsy or gastrin injection; and 9) patients who refused to participate in this study. The study was approved by the ethics committee of our institution and written informed consent was obtained from all subjects (UMIN00018967).

### Congo-red chromoendoscopy

After a clinical interview, a urea breath test was performed to check for current infection of *H. pylori*, and pentagastrin 6 μg/kg (Sigma-Aldrich, MO, USA) was administered intramuscularly followed by endoscopic inspection using a high-resolution tri-modal endoscope (EVIS-FQ260Z; Olympus, Japan). Approximately 15 minutes after the injection, Congo-red solution was sprayed in the whole stomach and any residual dye was carefully removed by suction. Its color change due to reaction with gastric acid was observed under endoscopic inspection and the distribution of AA and ANA was thoroughly and instantly evaluated under endoscopic observation (Figure 1A). After the extent of corpus atrophy was categorized by the Kimura-Takemoto classification, three biopsy specimens were obtained from non-neoplastic mucosa in each of AA and ANA of the corpus within 2 cm of their boundaries [8, 9].

### Mini-ussing chamber system

The *ex vivo* mucosal barrier function, sequential changes in resting potential (potential difference: PD) and mucosal permeability after the stimulation with ABC, *e.g.*, pH3 solution containing hydrochloric acid and a bile acid cocktail (200 μM glycolic acid, 200 μM taurocolic acid, 200 μM glycodeoxycholic acid, 200 μM deoxycholic acid, 200 μM taurodeoxycholic acid (Sigma-Aldrich)) were investigated using a mini-Ussing chamber system (Physiologic Instruments, CA, USA). The biopsy specimens were washed with oxygenated Krebs buffer, containing Na^+^ 152 mM, K^+^ 2.5 mM, Ca^2+^ 2.5 mM, Mg^2+^ 1.2 mM, Cl^−^ 136 mM, HCO_3_^−^ 25 mM, PO_4_^3-^ 1.2 mM, glucose 11 mM. The specimens were quickly clamped and fixed on the chamber with a 95% O_2_/5% CO_2_ mixed gas lift system. Cross-linked current electrodes and voltage electrodes were connected to both sides of the chamber, and the solution was connected to independent circulatory reservoirs [40].

### Electrophysiological measurements

After affixing samples, both sides of the lumen and base were perfused with pH 7.4 Krebs buffer for 30 minutes and the PD was measured using a measurement device; the change in potential when 10 μA current flowed was measured and impedance per unit area was calculated [41]. The lumen of the reservoir was perfused with the ABC solution, and the base with Krebs buffer. Mucosal impedance was measured every 15 minutes for a total of 120 minutes after the stimulation. Changes in mucosal impedance were expressed as the rate of change for impedance at the start of the measurement.

### Measurement of mucosal permeability

Fluorescein (376 kDa of molecular weight, Sigma-Aldrich) was low toxicity and high chromogenicity. Fluorescein at a 3 mg/ml concentration was administered at the dose of 150 μl to the lumen to measure the mucosal permeability. The solution of 200 μl each in the base and in the reservoir was collected every 15 minutes to maintain the amounts at an equal level. Finally, the concentration in the base side was measured using Fluoroscan Ascent (Thermo Fisher Scientific, Japan). Sequential changes in the fluorescein concentration were expressed as the rate of change compared to the start of the measurement.

### Histological evaluation

The biopsy specimens were scored for inflammation, activity, atrophy, and metaplasia using the updated Sydney system by a pathologist (Y.A.) blinded to any patient information [42].

### Immunohistological evaluation

The specimens were fixed in 10% buffered formalin and embedded in paraffin. After being autoclaved using Target Retrieval Solution (Dako, CA, USA), they were incubated with primary antibodies, including polyclonal anti-ZO1 antibody (1:1000, Abcam), polyclonal anti-claudin-12 antibody (1:1000, Abcam), polyclonal anti-snail antibody (1:1000, Abcam), overnight at 4° C. Thereafter, they were reacted with the second antibody, Vectastain ABC kit (1:200, Vectar laboratories, CA, USA), for 30 minutes. Binding with avidin-biotin labeling enzyme complex (Vectar laboratories) was performed followed by colorization in diamnobenzidine solution (Dako). Nuclei were stained with hematoxylin. The staining specificity was monitored in sections processed without the primary antibody (data not shown). Staining ability was evaluated using the scoring index reported by Yang *et al.* [43].

### Cell-lines

AGS (derived from human well-differentiated gastric cancer, ATCC) and MKN45 (derived from human poorly-differentiated gastric cancer, ATCC) cells were cultured in Dulbecco's modified Eagle's medium F-12 supplemented with 10% low endotoxin fetal bovine serum, antibiotics, and insulin-transferrin selenium X (Invitrogen, CA, USA) under a 5% CO2 environment. When 80–90% confluency was observed and, after starving, the culture was transferred to a serum-free medium containing 100 μM ABC for 24 hours. No cellular damage was observed by CellTiter 96 Aqueous One Solution Reagent (Promega, WI, USA).

### Western blot analysis

After protein was eluted by Mammalian Protein Extraction Reagent and a protease inhibitor (Thermo scientific, IL, USA), protein samples (10 μg/well) were separated by 4–12% NuPAGE Bis-Tris Gel (Invitrogen) and transcribed onto PVDF membranes (Millipore, MA, USA). After the blocking process, they were probed overnight at 4° C with primary antibodies, including polyclonal anti-ZO1 antibody (1:1000, Abcam), polyclonal anti-snail antibody (1:1000, Abcam), polyclonal anti-E-cadherin antibody (1:500, Abcam), polyclonal anti-Transforming growth factor-beta (TGFβ) antibody (1:1000, Abcam), polyclonal anit-ZEB1 antibody (1:2000, Bethyl Laboratories, TX, USA), monoclonal anti-Twist antibody (1:500, Abcam). Thereafter, they were incubated with secondary antibodies for 1 hour at room temperature. Protein detection by a detection reagent (GE healthcare, Japan) was observed by VersaDoc 5000MP (Bio-Rad Laboratories, CA, USA). Subsequently, the blots were stripped off and were reprobed with polyclonal anti-GAPDH antibody (1:1000, Cell Signaling). Densitometric analyses were performed using NIH image software. The concentration of each band was evaluated by the ratio to the GAPDH band in three separate experiments.

### RNA extraction

For the extraction of RNA and microRNA, RNeasy Mini Kit (Qiagen, CA, USA) and miRNeasy Mini Kit (Qiagen) were used in accordance with the manufacture's protocols.

### RT-qPCR

Following the treatment with DNaseI (Invitrogen), a reverse transcription reaction was performed using a Superscript VILO cDNA Synthesis kit (Invitrogen). As for microRNA, 10 ng of each RNA was used as template, and the primer for reverse transcription and Taqman Micro RNA Reverse Transcription Kit (Applied Biosystems, CA, USA) were used. Following the primers, ZO-1 (Hs01551861m1), GAPDH (Hs02758991g1), snail (Hs00195591m1), HMGA2 (Hs04397751m1), *let-7a* (00037), mir21 (002438), U6-snRNA (001973), were applied for RT-qPCR using a StepOnePlus system (Applied Biosystems). The PCR consisted of 40 cycles of reaction with 1 cycle being 95° C for 15 seconds, and 60° C for 1 minute after the initial degeneration at 95° C for 10 minutes. The expression levels were investigated using the comparative C_T_ method. GAPDH mRNA and U6-snRNA were used as the intrinsic control.

### Discovery phase

#### PCR array

RNA was extracted from frozen-stored biopsy specimens in the preceding study. cDNA was prepared by reverse transcription for 1 μg RNA sample and RCR was performed using a human tight junction RT^2^ PCR array (QIAGEN) in accordance with the manufacture's protocols. The target genes whose expression levels were at least 2-fold or below a 0.5-fold difference between AA and ANA were extracted as candidate genes.

#### Micro RNA microarray

MicroRNA was extracted from frozen-stored biopsy specimens in the preceding study. A Taqman microRNA array was used in accordance with the manufacture's protocol (Applied Biosystems). Target microRNAs whose expression levels were expressed at least 2-fold or below a 0.5-fold difference were selected as candidate microRNAs.

### Transfection of microRNAs

Lipofectamine RNAi MAX Reagent and Opti-MEM 1 reduced-serum medium (Invitrogen) were used for transfection with commercial plasmids, *let-7a* inhibitor (mirVana miRNA inhibitor, MH10050, Life Technologies, Japan), *let-7a* inhibitor negative control, *let-7a* miRNA mimic (MC10050), and *let-7a* mimic negative control in accordance with the manufacturer's protocol.

### MTT assay

AGSs were cultured with/without the ABC stimulation for 24 hours, followed by Cytoselect MTT Cell Proliferation Assay (Cell Biolabs, CA, USA) in accordance with the manufacturer's protocol.

### Cell infiltration assay

AGSs were cultured with/without the ABC stimulation for 24 hours, followed by Cytoselect 24-well cell invasion assay (Cell biolabs) in accordance with the manufacturer's protocol.

### Statistical analysis

Results were expressed as mean (standard deviation: S.D.) or (standard error: S.E.). Intergroup comparisons were tested using ANOVA, followed by a Chi-square test or a Mann-Whitney *U* test. Mixed effects model was used to test comparisons of sequential data that were measured repeatedly. Data were analyzed using JMP pro 12.0.1 (SAS Institute, NC, USA) and *P* values below 0.05 were considered significant.

## SUPPLEMENTARY MATERIALS FIGURES AND TABLES



## Abbreviations

GC: gastric cancer
H.pylori: Helicobacter pylori
AA: acid secretory area
ANA: acid non-secretory area
ABC: acidic bile acid cocktail
BA: bile acid
HMGA2: High mobility group A2
EMT: epithelial-mesenchymal transition
CRE: Congo-red chromoendoscopy
TJ: Tight junction
AJ: Adherence junction
RT-qPCR: Reverse Transcription-quantitative Polymerase Chain Reaction
TTER: transepithelial electric resistance
TGFβ: Transforming growth factor-beta
ZO-1: zonula occludens-1.
