# Supplementary material for: Acidic bile salts induces mucosal barrier dysfunction through let-7a reduction during gastric carcinogenesis after Helicobacter pylori eradication
Source: Oncotarget. 2018 Apr 6;9(26):18069–83. doi: 10.18632/oncotarget.24725 (PMC5915058; doi:10.18632/oncotarget.24725)
Supplement: Supplementary file 1 [file oncotarget-09-18069-s001.pdf]

Acidic bile salts induces mucosal barrier dysfunction through *let-7a* reduction during gastric carcinogenesis after *Helicobacter pylori* eradication

SUPPLEMENTARY MATERIALS

Supplementary Table 1: The background data and demographics of the subjects

N		18
Age (mean (S.D.))		74.0 (7.6)
Gender (Male/ Female)		15/3
Duration after eradication (measn (S. D.) months)		74.8 (51.5)
Mucosal atrophy in Endoscopic findings		
White light	C1-2/ C3-O1/ O2-3	2/ 6/ 10
Congo-red endoscopy	C1-2/ C3-O1/ O2-3	3/ 7/8
Gastric Cancer		
location	U/M/L	3/ 1/ 14
Size (mean (S.D.) mm)		12.5 (13.9)
Macroscopic type	Protruded/ depressed	2/ 16
Tumor staging	T1a/ T1b-	15/ 3
Histological type	Wel-mod/ por-sig/ mixed	18/0/0

The endoscopic findings of tumor location, macroscopic type and tumor size were classified based on the classification for Gastric Cancer (Japanese Classification of Gastric Carcinoma - 2nd English Edition. Gastric cancer). The extent of corpus atrophy was assessed and categorized by the Kimura-Takemoto classification. (S.D.; standard deviation).

Supplementary Table 2: The updated Sydney system for post-eradication gastric mucosa

		Acid non-secreting area	Acid secreting area	p-value
Inflammation	0/1/2/3	0/4/14/0	0/17/1/0	P<0.0001
Activity	0/1/2/3	18/0/0/0	18/0/0/0	-
atrophy	0/1/2/3	2/1/1/14	16/1/0/1	P<0.0001
metaplasia	0/1/2/3	1/2/0/15	15/2/0/1	P<0.0001

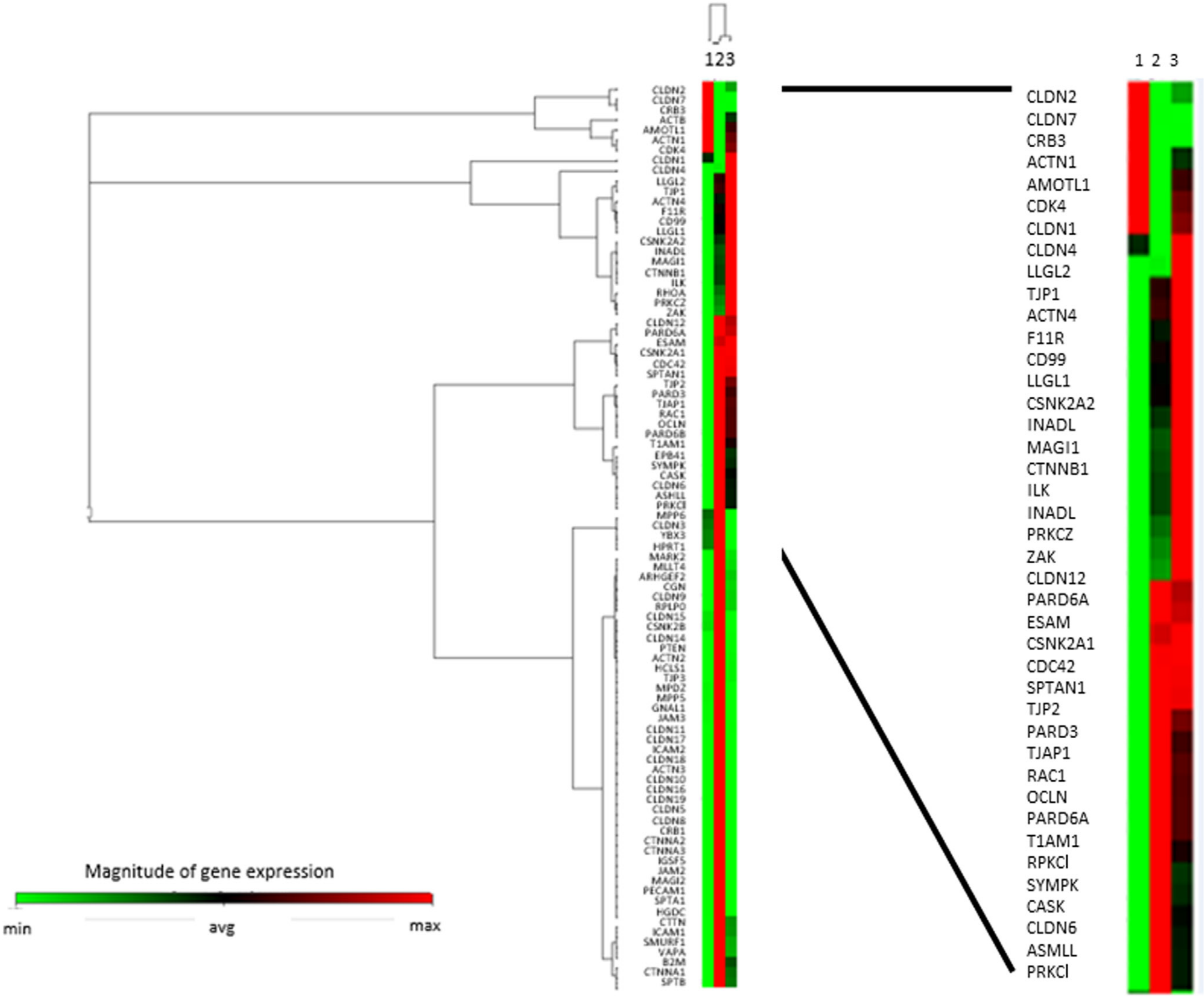

Supplementary Figure 1: The similarity of the phenotype of TJ gene expressions in AGS, rather than MKN45, to that of human gastric mucosal tissue after H.pylori eradication.
